# Insights into Nasal Carriage of *Staphylococcus aureus* in an Urban and a Rural Community in Ghana

**DOI:** 10.1371/journal.pone.0096119

**Published:** 2014-04-23

**Authors:** Beverly Egyir, Luca Guardabassi, Joseph Esson, Søren Saxmose Nielsen, Mercy Jemima Newman, Kennedy Kwasi Addo, Anders Rhod Larsen

**Affiliations:** 1 Department of Microbiology and Infection Control, Statens Serum Insitut, Copenhagen, Denmark; 2 Department of Veterinary Disease Biology, Faculty of Health and Medical Sciences, University of Copenhagen, Copenhagen, Denmark; 3 Bacteriology Department, Noguchi Memorial Institute for Medical Research, Accra, Ghana; 4 Microbiology Department, University of Ghana Medical School, Accra, Ghana; 5 Department of Large Animal Sciences, Faculty of Health and Medical Sciences, University of Copenhagen, Copenhagen, Denmark; National Institutes of Health, United States of America

## Abstract

The epidemiology of *Staphylococcus aureus* in the community in Ghana was never investigated prior to this study. The aims of the study were: i) to assess prevalence of nasal *S. aureus* carriage in Ghanaian people living in an urban and a rural area, and ii) to identify phenotypic and genotypic traits of strains isolated from the two communities. Nasal swabs were collected from healthy individuals living in an urban community situated in the suburb of the capital city, Accra (n = 353) and in a rural community situated in the Dangme-West district (n = 234). The overall prevalence of nasal carriage was 21% with a significantly higher prevalence in the urban (28%) than in the rural community (11%) (p<0.0001). The levels of antimicrobial resistance were generally low (<5%) except for penicillin (91%) and tetracycline (25%). The only two (0.3%) MRSA carriers were individuals living in the urban area and had been exposed to hospitals within the last 12 months prior to sampling. Resistance to tetracycline (p = 0.0009) and presence of Panton-Valentine leukocidin (PVL) gene (p = 0.02) were significantly higher among isolates from the rural community compared to isolates from the urban community. Eleven MLST clonal complexes (CC) were detected based on *spa* typing of the 124 *S. aureus* isolates from the two communities: CC8 (n = 36), CC152 (n = 21), CC45 (n = 21), CC15 (n = 18), CC121 (n = 6), CC97 (n = 6), CC30 (n = 5), CC5 (n = 5), CC508 (n = 4), CC9 (n = 1), and CC707 (n = 1). CC8 and CC45 were less frequent in the rural area than in the urban area (p = 0.02). These results reveal remarkable differences regarding carriage prevalence, tetracycline resistance, PVL content and clonal distribution of *S. aureus* in the two study populations. Future research may be required to establish whether such differences in nasal *S. aureus* carriage are linked to socio-economic differences between urban and rural communities in this African country.

## Introduction

*Staphylococcus aureus* is carried by 20–30% of the human population, defined as persistent carriers, whereas the remaining part of the population does not carry or is only transiently colonized by this opportunistic pathogen [1]. Colonization of the anterior nares is a recognized risk factor for subsequent *S. aureus* infection, although carriers generally have a better prognosis [2]. Risk factors for colonization include young age, male sex, underlying comorbidities, hospitalization and exposure to livestock [2]–[5].

Data on *S. aureus* nasal carriage in the community are largely based on developed countries [6]–[13] and reports from sub-Saharan African countries are very limited [14]–[17]. High frequencies (>55%) of strains carrying Panton-Valentine leukocidin (PVL), a toxin associated with community-acquired methicillin-resistant *S. aureus* (CA-MRSA) [18], [19], have been reported in Mali [14] Gabon [16], [17] and Nigeria [20], [21], suggesting that a common CA-MRSA clone associated with PVL (sequence type ST152) could have evolved from a methicillin-susceptible variant of this clone originating from the African continent [14], [16].

We recently reported a significant difference in nasal carriage prevalence between inpatients (14%) and staff (23%) at the largest hospital in Ghana [22]. This and a subsequent study on clinical *S. aureus* isolates [23] showed high frequency of PVL-positive isolates (23–60%) with predominance of t355 (CC152) and t084 (CC15) in Ghana. The epidemiology of *S. aureus* among healthy people in the Ghanaian community, however, remains unknown.

The aims of this study were: i) to assess prevalence of nasal *S. aureus* carriage in Ghanaian people living in an urban and a rural area, and ii) to identify phenotypic and genotypic differences between strains isolated from the two communities. For this purpose, isolates obtained from the two communities were compared with respect to antimicrobial susceptibility, PVL gene content and genetic background.

## Materials and Methods

### Ethical Approval

Ethical clearance was obtained from the University of Ghana Medical School Ethical and Protocol Review Board (reference no. MS-Et/M.8 - P.4.4/2010–11). The consent document was signed or thumb printed by participants or guardians (on behalf of children) and a witness. The consent request was interpreted in the local dialect by the researcher to participants or guardians who could not comprehend the English language. The Ethical and Protocol Review Board approved the consent documents and procedures.

### Study Design, Area and Population

**Figure 1:**
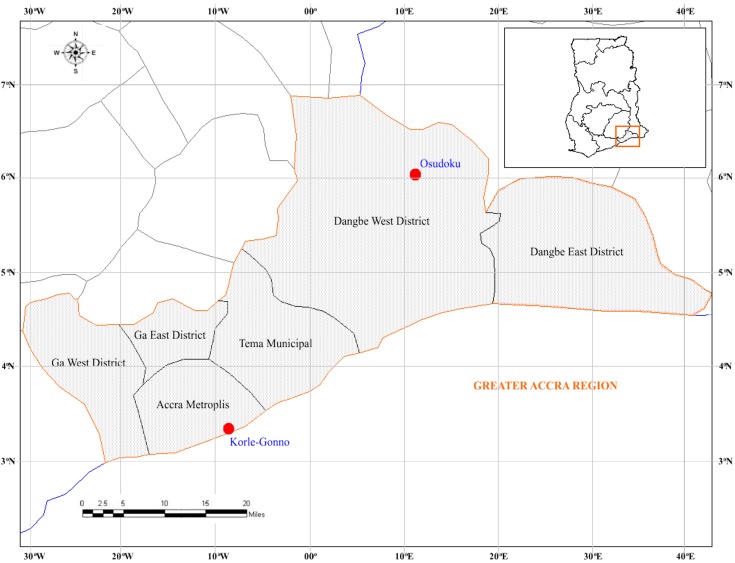
Map showing the location of the two communities in Ghana.

A cross-sectional-type study was conducted between May 2011 and April 2012 at two communities located in Southern Ghana: Korle Gonno (urban) and Osudoku (rural); approximately 102.5 kilometres (63.7 miles) apart. Korle Gonno is a densely populated and overcrowded suburb of Accra Metropolis District with an approximate population of 60,000 inhabitants. Korle gonno is primarily, a fishing community but has a market and beach resorts which attracts clients from various parts of Ghana. This urban community has easy access to pharmacies and to the largest teaching hospital in Ghana, which serve the majority of the population in Ghana. Osudoku is a rural area situated in the Dangme-West district with a low population size of 18,972 inhabitants. Agriculture, animal husbandry and hunting are the prevalent economic activities. Access to healthcare facilities is limited in this remote area. The two communities are located in the Greater Accra region of Ghana (Figure 1).

### Sample Collection and Isolation of *S. aureus*

Apparently healthy persons were enrolled based on their consent to participate in the study. Nasal samples were taken by rotating sterile cotton swabs in both anterior nares. Using a standardised questionnaire, information regarding the following demographic data and risk factors were obtained from each participant: age, sex, exposure to hospitals (i.e. visit as an outpatient or hospitalization) and self-reported antimicrobial therapy within the last 12 months, self-reported diseases such as eczema or diabetes and presence of a healthcare worker in household.

Pre-enrichment of nasal swabs was performed in 5 mL Muller Hinton broth supplemented with 6.5% NaCl, (Oxoid, Ltd., Basingstokes, UK) within 24 hrs after sampling and incubated at 37°C for 24 hrs and plated on 5% sheep blood agar (Oxoid, Ltd., Basingstokes, UK). *S. aureus* isolates were identified phenotypically by colony morphology, haemolysis, catalase test, Gram staining and confirmed by tube coagulase, slidex staphplus test (bioMérieux, Marcy l′Etoil, France) and PCR identification of the *spa* gene [24].

### Antimicrobial Susceptibility Testing

Susceptibility testing of 11 antimicrobial agents was done according to EUCAST guidelines (www.eucast.org) as described elsewhere [22]. MRSA isolated were tested for glycopeptide resistance as previously described [25]. Multidrug resistance (MDR) was defined as resistance to three or more distinct antimicrobial classes [26].

### Molecular Characterization

Detection of *spa*, *mecA* and *pvl* genes was performed according to standard methods [24], [27]. For all isolates, *spa* types and MLST CCs were assigned using BioNumerics V6.5 (Applied Maths, Sint-Martens-Latem Belgium) with the *spa* (http://www.spaserver.ridom.de) plug in. Multi-Locus Sequence Typing (MLST) [28] and Staphylococcal cassette chromosome *mec* (SCC*mec*) typing by multiplex PCR assays 1 and 2 [29] were done limited to MRSA isolates.

### Statistical Analysis

Data were analyzed using the glm-function in R version 2.15.2 (R Development Core Team, 2012). Possible associations of *S. aureus* nasal carriage with age, sex and the other four risk factors investigated by the questionnaire (exposure to hospital environment, self-reported antimicrobial therapy, self-reported disease, and health worker in household) were assessed by logistic regression analysis. The confounding effects were assessed for each factor through assessment of the effect of the parameter estimates by offering the factors individually and assessing the effect from this. Furthermore, two-way interactions were assessed. For analytical purposes, community-associated (CA) *S. aureus* was defined as isolates from participants without previous exposure to the hospital environment, whereas community-onset (CO) *S. aureus* referred to isolates from participants with a history of hospital exposure within the last 12 months [30]. Model fit was assessed using the Pearson *Χ^2^*-statistics to judge unexplained extra-binomial variation. Relative risks of significant parameters were subsequently estimated. The two-sample z-test was used to compare the proportion of strains resistant to the different antimicrobials in the two areas. Furthermore, the association of genotypes (*spa* type, CC-groups, PVL-positivity) to sex and source (community) were determined using the Fisher exact test. Only the four most prevalent genotypes with more than 10 observations were included in this analysis.

## Results

### Study Population and *S. aureus* Carriage

A total of 587 nasal swabs were obtained from the two study areas: 353 (60%) from Korle Gonno (urban) and 234 (40%) from Osudoku (rural). Demographic characteristics of the two study populations are shown in Table 1. The participants included 370 (63%) females and 217 (37%) males, with an overall mean age of 32 years (age range: 1–115 years). Among the 587 study participants, 216 (36.8%) indicated exposure to the hospital environment within the last 12 months (Table 1). Forty-one (7%) reported recent usage of antimicrobial agents, including penicillin (n = 34, 6%); ciprofloxacin (n = 5, 0.9%) and tetracycline (n = 2, 0.3%).

**Table 1:** Demographic characteristics of the study participants from the rural community in Osudoku (OS) and the urban community in Korle-gonno (KG), Ghana, 2011–2012.

Characteristic	Category	OS (N = 234)	KG (N = 353)	Total (N = 587)	p-value
		n (%)	n (%)	n (%)	
Age (mean/sd)		34.5 (23.2)	29.7 (20.6)	31.6 (21.8)	0.0085
Sex	Female	140 (59.8)	230 (65.2)	370 (63.0)	
	Male	94 (40.2)	123 (34.8)	217 (37.0)	0.2218
EH	No	132 (56.4)	239 (67.7)	371 (63.2)	
	Yes	102 (43.6)	114 (32.3)	216 (36.8)	0.0071
SRD	No	192 (82.1)	338 (95.8)	530 (90.3)	
	Yes	42 (17.9)	15 (4.2)	57 (9.7)	<0.0001
SRAT	No	209 (89.3)	337 (95.5)	546 (93.0)	
	Yes	25 (10.7)	16 (4.5)	41 (7.0)	0.0069
HWHH	No	227 (97.0)	284 (80.5)	511 (87.1)	
	Yes	7 (3.0)	69 (19.5)	76 (12.9)	<0.0001

sd, standard deviation; EH: exposure to hospitals; SRD: Self-reported disease; SRAT: self-reported antimicrobial therapy; HWHH: healthcare worker in household.

*S. aureus* was identified in 21% (124/587) of the nasal swabs cultured. Among the 124 carriers, 98 (79%) resided in the urban area and 26 (21%) in the rural area. (Table 2). The nasal carriage prevalence was significantly higher at Korle Gonno (urban) (98/353; 28%) compared to Osudoku (rural) (26/234; 11%) (p<0.0001), resulting in a 2.5 (95% CI: 1.7–3.7) higher risk of being a carrier in the urban area. No confounding effects were detected. The proportions of carriers in the age groups 0–9, 10–19, 20–29, 30–39, 40–49, 50–59, 60–69 and >70 years were 19%, 29%, 19%, 17%, 20%, 20%, 19% and 18%, respectively. No statistical differences in nasal carriage of *S. aureus* and MRSA were detected relating to age, sex, exposure to hospitals, self reported disease, self reported antimicrobial therapy and health worker in household.

**Table 2:** Characteristics of *S. aureus* non-carriers and carriers in the two Ghanaian communities, 2011–2012.

Characteristic	Category	Non-carriers (N = 463)	Carriers (N = 124)	Total (N = 587)	p-value
		n (%)	n (%)	n (%)	
Age (mean/sd)		32.1 (22.1)	29.8 (20.3)	31.6 (21.8)	0.2812
Sex	Female	290 (62.6)	80 (64.5)	370 (63.0)	
	Male	173 (37.4)	44 (35.5)	217 (37.0)	0.7790
Community	OS	208 (44.9)	26 (21.0)	234 (39.9)	
	KG	255 (55.1)	98 (79.0)	353 (60.1)	<0.0001
EH	No	285 (61.6)	86 (69.4)	371 (63.2)	
	Yes	178 (38.4)	38 (30.6)	216 (36.8)	0.1350
SRD	No	414 (89.4)	116 (93.5)	530 (90.3)	
	Yes	49 (10.6)	8 (6.5)	57 (9.7)	0.2266
SRAT	No	430 (92.9)	116 (93.5)	546 (93.0)	
	Yes	33 (7.1)	8 (6.5)	41 (7.0)	0.9491
HWHH	No	405 (87.5)	106 (85.5)	511 (87.1)	
	Yes	58 (12.5)	18 (14.5)	76 (12.9)	0.6633

OS: Osudoku (rural); KG: Korle-gonno (urban); EH: exposure to hospitals; SRD: self-reported disease; SRAT: self-reported antimicrobial therapy; HWHH: healthcare worker in household.

### Prevalence of Antimicrobial Resistance

Among the 124 *S. aureus* isolates, 113 (91%) were resistant to penicillin and 31 (25%) to tetracycline. Resistance to erythromycin, fusidic acid, norfloxacin and cefoxitin was below 5% (Table 3). The level of resistance to tetracycline was significantly higher in Osudoku (50%) compared to Korle Gonno (18%) (p = 0.0009), whereas there were no detectable differences for the remaining antimicrobials. All isolates were susceptible to clindamycin, trimethoprim-sulphamethoxazole, gentamicin, rifampicin, mupirocin, and linezolid. Seven (6%) isolates were MDR, with penicillin-tetracycline-norfloxacin (n = 5) being the predominant resistance profile.

**Table 3:** Percentage antimicrobial resistance in *S. aureus* isolated from nasal carriers in two Ghanaian communities, 2011–2012.

Antimicrobial Agent	OS (N = 26)	KG (N = 98)	Total (N = 124)
	n (%)	n (%)	n (%)
Penicillin	23 (88.4)	90 (91.8)	113 (91.0)
Tetracycline	13 (50.0)	18 (18.4)	31 (25.0)
Fucidic acid	1 (3.8)	2 (2.0)	3 (2.4)
Cefoxitin	0 (0.0)	2 (2.0)	2 (1.6)
Erythromycin	1 (3.8)	1 (1.0)	2 (1.6)
Norfloxacin	0	6 (6.1)	6 (4.8)

OS: Osudoku (rural), KG: Korle Gonno (urban). No resistance was detected in both areas for Trimethoprim Sufamethozaxole, Clindamycin, Gentamicin, Rifampicin Mupirocin and Linezolid.

Only two isolates (1.6%; 2/124) were resistant to cefoxitin and confirmed to be MRSA by *mecA* PCR. Both MRSA isolates were susceptible to all non-beta lactam antimicrobials tested and originated from participants from Korle Gonno with a history of exposure to hospital environment within the last 12 months: a 60 years old male who declared to have taken antihypertensive drugs and a 27 years old female who reported usage of penicillin and other drugs she could not identify.

### Genetic Diversity of *S. aureus* Isolates

The most prevalent among the 46 *spa* types found were t355, (CC152, 15%), t084 (CC15, 11%), t10519 (CC8, 10%), and t008 (CC8, 9%). Thirty-one singletons, including 10 new *spa* types (t10832, t10834, t10835, t10839 to t10845) were found among the isolates. Based on *spa* typing the following CCs were identified among the isolates: CC8 (n = 36), CC152 (n = 21), CC45 (n = 21), CC15 (n = 18), CC121 (n = 6), CC97 (n = 6), CC30 (n = 5), CC5 (n = 5), CC508 (n = 4), CC9 (n = 1), and CC707 (n = 1). Distributions of the most frequent *spa* types (t355, t084, t10519 and t008) and CCs (CC8, CC152, CC45, CC15 and CC121) differed significantly (p = 0.04 and p = 0.014, respectively) between Osudoku and Korle-gonno, and were not influenced by age and sex.

Thirty-four (27%) isolates were PVL-positive (Table 4), the majority of which belonged to CC152 (n = 15), CC15 (n = 7), CC30 (n = 3), CC121 (n = 3) and CC5 (n = 2). Among the four most frequent *spa* types, PVL was associated to t355 (CC152) (38%) and t084 (CC15) (15%) (p<0.01), while it was not detected in t008 (CC8) and t10519 (CC8). There was a two times increased risk of carrying a PVL-positive *S. aureus* in Osudoku (12/26, 46%) compared to Korle Gonno (22/98, 22%) (p = 0.03, 95% CI: 1.2–3.7). The two MRSA isolates were PVL-negative, displayed the same *spa* type (t5132) associated to ST508, and carried SCC*mec* type V.

**Table 4:** Distribution of *spa* types and PVL within each *Staphylococcus aureus* clonal complex (CC) detected in Osudoku (rural) and Korle Gonno (urban) communities in Ghana, 2011–2012.

CC	Distribution of *spa* types		PVL frequency (N = 34)
	Osudoku (N = 26)	Korle Gonno (N = 98)	
CC5	Not detected	t10839(1), t311(3), t071(1)	2/5
CC8	t1476(3), t10519(1)	t008(11), t1476(5), t10519(12), t10842(1) 10844(1), t197(1), t304(1)	0/36
CC9	Not detected	t2700(1)	0/1
CC15	t084(5)	t7568(1), t084(9), t346(1), t10843(1), t10845(1)	7/18
CC30	t3194(1), t021(1)	t021(2), t363(1)	3/5
CC97	t359(4)	t359(1)	2/5
	Not detected	t044(1)	0/1
CC45	t861(1)	t2771(3), t5602(3), t6038(2), t1996(1) t065(1), t10834(1), t10840(1),t10841(1), t1510(1) t3986(1), t861(2), t939(1), t2784(1) t8453(1)	1/21
CC121	t159(1), t2304(1)	t091(2), t4499(1), t645(1)	3/6
CC152	t454(1), t355(6), t10835(1)	t454 (1), t355(12)	15/21
CC508	Not detected	t5132(2), t10832(1) t6694 (1)	0/4
CC707	Not detected	t1458(1)	1/1

CC152 occurred more frequently among CA isolates than among CO isolates (p = 0.01), whereas CC8 was more common among CO isolates (p = 0.04) No other significant differences were observed in the occurrence of CCs among CA and CO isolates (Table 5).

**Table 5:** Distribution of *spa* types among community-onset (CO) and community-associated (CA) *S. aureus* isolates from the two Ghanian communities, 2011–2012.

CC	Total no of isolates, N = 124	CO isolates	N = 38	CA isolates	N = 86
	n (%)		n (%)		n (%)
CC5	5 (4.0)	t071(1), t10839(1), t311(2)	4 (10.5)	t311(1)	1 (1.2)
CC8	36 (29.0)	t008(4), t10519(5), t10842(1), t1476(3), t304(1)	14 (36.8)	t008(7), t10519(8), t10844(1), t1476(5), t197(1)	22 (25.6)
CC9	1 (0.8)	t2700(1)	1 (2.6)	-	0 (0.0)
CC15	18 (14.5)	t084(3), t10843(1), t10845(1) t7568(1)	6 (15.8)	t084(11), t346(1)	12 (13.9)
CC30	5 (4.0)	t021(2)	2 (5.2)	t021(1), t3194(1), t363(1)	3 (3.5)
CC45	21 (16.9)	t10841(1), t6038(1),	2 (5.2)	t065(1),t10834(1), t10840(1), t1510(1), t1996(1), t2771(3), t2784(1), t3986(1),t5602(3), t6038(1), t8453(1), t861(3), t939(1)	19 (22.1)
CC97	6 (4.8)	t359(1), t044(1)	2(5.2)	t359(4)	4 (4.7)
CC121	6 (4.8)	t4499(1)	1 (2.6)	t091(2), t159(1), t2304(1), t645(1)	5 (5.8)
CC152	21 (16.9)	t10835(1), t454(2)	4 (10.5)	t355(17)	17 (19.7)
CC508	4 (3.2)	t5132(2)	2 (5.4)	t10832(1), t6694(1)	2 (2.3)
CC707	1 (0.8)	-	0 (0.0)	t1458(1)	1 (1.2)

## Discussion

We investigated the prevalence of nasal *S. aureus* carriage among healthy individuals in two communities representing urban and rural areas in Ghana. The overall prevalence of 21% is within the range (14–33%) of those previously reported in other African countries such as Nigeria [15], [31] and Gabon [16], [17]. Based on these data, the frequencies of *S. aureus* in African communities seems to resemble those obtained in communities of developed countries [6], [10], [12], even though carriage has been hypothesized to vary between different races [6], [32]. Interestingly, the frequency of nasal carriage among people from the urban community was significantly higher than among people from the rural community. This is apparently in contrast with recent study in Gabon, where *S. aureus* carriage was higher (37%) in subjects from rural areas than in subjects from semi-urban areas (21%) [17]. The different *S. aureus* carriage prevalence observed between urban and rural dwellers might be linked to specific socio-economic differences between the two communities. For example, inhabitants in the urban area live in overcrowded settlements compared to those in the rural area.

The prevalence of antimicrobial resistance was generally low with the only exception of penicillin resistance, which is widespread in human isolates of *S. aureus* worldwide [15], [32], [33]. One exception to this general rule is provided by a remote community of Pigmies in Gabon, where high susceptibility to penicillin (>60%) has recently been reported, probably as a consequence of a limited usage of antimicrobial agents in this population [16] . The significantly higher frequency of tetracycline resistance observed in this study in the rural community compared to the urban community could be correlated to veterinary use of tetracycline since this antibiotic is commonly used in livestock farming in Ghana [34], [35]. However, none of the *S. aureus* lineages found in this rural community is known to be livestock-associated. Possible associations with the use of tetracycline in this community could not be determined since regional data on antimicrobial consumption are not available. Antimicrobial usage might be higher than indicated by our questionnaire study considering the high rate of self-medication [36] and widespread usage of antimicrobial agents in Ghana and most African countries [37]–[39].

Carriage of MRSA (2/578) in the Ghanaian community was low (0.3%) and exclusively associated to urban participants recently exposed to hospitals. This is in line with the low prevalence (0.9%) of MRSA nasal carriage previously reported among inpatients and staff at the hospital in Ghana [22] and the lack of MRSA detection by previous studies conducted on remote communities in Africa [16]. High prevalence (27.5%) of presumptive MRSA was reported in a community study in Nigeria, a neighboring country [15]. It should however be noted that presumptive MRSA detected in the Nigerian study were not confirmed genotypically by PCR detection of *mec*A.

Both MRSA isolates detected in the urban community belonged to a rare *spa* type (t5132) associated to ST508. The two MRSA-positive individuals lived in the urban area. As they shared isolates with the same uncommon *spa* type, it cannot be excluded that the two cases were epidemiologically related. ST508 is a single locus variant of the Berlin epidemic clone ST45 [40]. Interestingly, ST508 was isolated among methicillin-susceptible isolates in the previous study on hospital carriage in Ghana [22], among clinical and community isolates in Gabon [17], [41] and Nigeria [20], [42], indicating that a reservoir of ST508 exists in these parts of Africa. The ST508 MRSA isolates shared the SCC*mec*V element often associated with CA-MRSA isolates [43], [44] but lacked PVL [43]. Two out of six MRSA isolated in the previous hospital carriage study in Ghana also harbored SCC*mec* V, whereas the remaining four isolates contained SCC*mec* types I or IV [22].

The population structure of *S. aureus* isolated from the two communities in Ghana was heterogeneous. Specific *spa* types such as t355 and t084 were frequent in this study as well as in the previous studies conducted among nasal carriers at the hospital in Korle Gonno (Korle Bu Teaching Hospital) [22] and clinical isolates from Ghanaian health care institutions [23], indicating that these lineages (CC152 and CC15, respectively) are widespread in Ghana. The predominant distribution of CC152 and CC15 in this study resembles what has been found in a nasal carrier study in Mali [14] and Gabon [41]. Some of the most common CCs (CC152, CC15 and CC8) and *spa* types (t355, t084 and t008) were commonly isolated from nasal carriers in the previous hospital study [22]. Within CC8, t10519 was relatively common in this study, especially in the urban community of Korle Gonno (12%), but was not detected in the local hospital [22], suggesting that this *spa* type is mainly restricted to the local community and has limited ability to spread inside the hospital. On the contrary, CC1 and CC88, which were detected among the nasal carriers in the hospital study in Ghana [22], were not detected in the community, indicating that these lineages may be associated with hospital environments in Ghana.

Noteworthy, t044, a widely disseminated *spa* type associated with MRSA ST80 in Middle East [45] and Europe [46], was isolated from an urban participant as MSSA. Further characterization by whole genome sequencing (data not shown) showed that this strain belonged to another lineage (ST669, CC97) not related to CC80. This phenomenon has been described for other *spa* types. For example, t037 has been associated with both CC8 (ST239) and CC30, probably as a result of chromosomal recombination within the variable region of *spa* [47], [48]. Thus, although good correlation between *spa* types and MLST CCs has been previously reported [48], the fact that MSSA isolates were only characterized by *spa* typing may be regarded as a limitation of the study, since this approach may not lead to accurate clonal identification at the ST and CC levels. However, this is a minor limitation as it does not influence the genotypic and phenotypic differences observed between the two communities.

The high frequency (27%) of PVL-positive isolates was anticipated as a similar trend was seen among nasal isolates from the Korle Bu hospital [22], clinical isolates from health care institution in Ghana [23] and in other African studies [14], [16], [20], [42]. The higher prevalence of PVL-positive isolates in the rural community mirrors the finding in a remote (rural) Gabonese community compared to urban populations [16] and is likely to reflect differences in the distribution of genetic lineages between the two communities.

To the best of our knowledge, this is the first study investigating carriage prevalence, antimicrobial resistance and clonal distribution of *S. aureus* in the Ghanaian community. The study reveals marked differences between an urban and a rural area with respect to carriage rate, tetracycline resistance, PVL content and clonal distribution. Even though the distribution of *S. aureus* clones differed significantly between the two communities, the difference was small and may not necessarily be biologically relevant. Further studies may be required to establish the socio-economic factors that may be responsible for the geographical differences observed between these two Ghanaian communities.
